# Fecal microbial signatures of healthy Han individuals from three bio-geographical zones in Guangdong

**DOI:** 10.3389/fmicb.2022.920780

**Published:** 2022-08-08

**Authors:** Litao Huang, Liting Deng, Changhui Liu, Enping Huang, Xiaolong Han, Cheng Xiao, Xiaomin Liang, Huilin Sun, Chao Liu, Ling Chen

**Affiliations:** ^1^Guangzhou Key Laboratory of Forensic Multi-Omics for Precision Identification, School of Forensic Medicine, Southern Medical University, Guangzhou, China; ^2^The First Affiliated Hospital of Guangdong Pharmaceutical University, Guangzhou, China; ^3^Guangzhou Forensic Science Institute, Guangzhou, China

**Keywords:** forensic medicine, feces, gut microbiome, 16S rRNA gene sequencing, Guangdong Han individuals

## Abstract

Important forensic evidence traced from crime scenes, such as fecal materials, can help in the forensic investigation of criminal cases. Intestines are the largest microbial pool in the human body whose microbial community is considered to be the human “second fingerprint”. The present study explored the potential for community characteristics of gut microbes in forensic medicine. Fecal microbiota profiles of healthy individuals from three representative Han populations (Guangzhou, Shantou and Meizhou) in Guangdong Province, China were evaluated using High-throughput sequencing of V3-V4 hypervariable regions of the 16SrRNA gene. Results of the present study showed that at the genus level, Shantou, Guangzhou, and Meizhou behaved as Enterotype1, Enterotype2, and Enterotype3, which were mainly composed of Bacteroides, Prevotella, and Blautia, respectively. Based on OTU abundance at the genus level, using the random forest prediction model, it was found that there might be potential for distinguishing individuals of Guangzhou, Meizhou, and Shantou according to their fecal microbial community. Moreover, the findings of the microbial community of fecal samples in the present study were significantly different from that of saliva samples reported in our previous study, and thus it is evident that the saliva and feces can be distinguished. In conclusion, this study reported the fecal microbial signature of three Han populations, which may provide basic data for the potential application in forensic practice, containing body fluid identification, and geographical inference.

## Introduction

Human beings live in a world full of microbes, and co-evolution, co-adaptation as well as co-dependence are the relationships between them and indigenous microbiota (Turnbaugh et al., 2007; Blaser and Falkow, 2009). Microorganisms exist in many sites of the human body mainly in the intestines. They also have a profound influence on human physiological metabolism and nutrition regulation (Hooper and Gordon, 2001). The human intestinal tract, a nutrient-rich microenvironment, carries 100 trillion (10^14^) bacteria which are about 10 times more than the number of human cells (Hooper et al., 2002; Bäckhed et al., 2005). The colon is the main contributor to the total number of bacteria in the entire intestine with a density close to 10^11^-10^12^cells/ml (Ley et al., 2006; Sender et al., 2016). Non-invasive fecal samples (the part in the middle of feces that is not in contact with the air and the ground) are usually considered representative of colonic microorganisms for they are easy to obtain and do not harm the subject (Davenport et al., 2017). Next-generation sequencing (NGS) using 16SrRNA gene sequence analysis overcomes the shortcomings that most traditional microorganisms cannot be cultivated and performed much deeper microbial community analysis at a low cost (Weinstock, 2012).

Feces have been specified as the important evidence for specific crimes, including burglary, robbery, and sexual cases. Particularly, in anal sexual assault cases, fecal traces of the victim may be left on a condom at the crime scene (Johnson et al., 2005). When analyzed, the microbial community in feces may help in individual identification and tracing the source of tissues and body fluids. Quaak et al. distinguished individuals by researching the microbial profiles generated in fecal samples from 35 healthy volunteers of different ages. It was then proposed that individual identification can be carried out by applying the fecal microbial profile to the increase evidence value of the trace when there was no or only part of human STR in fecal samples (Quaak et al., 2017). Microarray was also performed to analyze 175 samples from healthy individuals, successfully distinguishing and identifying the oral cavity, feces, and skin samples. The study noted that it might be beneficial for presenting important corroborating evidence for the scene left by the victim and/or suspect, aiding in the reconstruction of a case process (Quaak et al., 2018).

Recent studies have shown that the human intestinal microbial community is not only affected by the host's own factors, but also by external factors (Wen and Duffy, 2017). In general, geography and environment have shown the main influence on intestinal microbes (He et al., 2018; Rothschild et al., 2018). Guangdong Province is located in the southernmost part of mainland China and is an important heritage site of Lingnan culture. Lingnan Han groups, consisting of the Guangfu, Hakka, and Chaoshan, account for a majority of Han people in Guangdong. They have a unique culture in terms of language, customs, and living habits. For instance, Guangfu people speak Cantonese, cook Cantonese cuisine, and live mainly in the Pearl River Delta area of Guangdong. Further, Hakkas people are concentrated in northern Guangdong, mainly Hakka dialect and Hakka cuisine, together with Chaoshan people living in eastern Guangdong have their own Chaoshan dialects and Chaoshan cuisine (Wang et al., 2010; Du et al., 2019). Guangdong's three Han characteristic population was recognized as a branch of Han Chinese, and the gut microbiome characterization and forensic potential of these three groups are poorly defined or still need to be explored. The current study aimed to reveal the differences in fecal microbiota between the groups. Indigenous Han individuals from Guangzhou, Meizhou, and Shantou were selected as the representative of Guangfu, Hakka, and Chaoshan individuals, respectively. The fecal samples were collected and characterized through high-throughput sequencing of the samples in the V3-V4 region of the 16SrRNA gene. The prospect of forensic application of fecal microbiota was valued.

## Materials and methods

### Sample collection

This study was approved by the Biomedical Ethics Committee of Southern Medical University, Guangzhou, China. After obtaining informed consent, a total of 59 fecal samples were collected from healthy Han individuals (aged between 16 and 62) who had lived in Guangzhou, Meizhou, and Shantou for more than three generations in Guangdong Province, China. A total of 19, 20, and 20 samples from people in Guangzhou, Meizhou, and Shantou, respectively were collected. Participants were balanced by age and sex, divided into age1 (16–32 years old) and age2 (33–62 years old) groups, male and female groups. The participants received adequate training and guidance on the sample collection process before fecal collection and one sample was then collected per participant. The exclusion criteria were (1) participants who reported antibiotic use/other treatments within 3 months. (2) participants were diagnosed with any inflammation-related bowel disease or gastrointestinal disease within 3 months. (3) participants who lived <1 year or left the province within 1 month. According to the above criteria, a total of 59 healthy individuals from the three regions were included, and all fecal samples collected were named “F” (Guangzhou sample numbered from 1 to 19, Shantou sample numbers were from 20 to 39, Meizhou sample numbers were from 40 to 59). The participants used a sterile spoon to dig out a fallen scoop (about 3–5g) of fecal samples, collected them in a sterile plastic container, and immediately stored them in a refrigerator at-80°C in the laboratory awaiting extraction of the fecal bacterial genomic DNA.

### DNA extraction, PCR amplification, and sequencing

Bacterial genomic DNA in the samples was extracted using QIAamp DNA Stool Mini Kit (QIAGEN, Hilden, Germany), according to the manufacturer's instructions. The concentration and purity of DNA were quantified by using an ultraviolet spectrophotometer and DNA extraction quality is checked by 1% agarose gel electrophoresis. Qualified DNA samples were amplified using bacterial 16S rRNA corresponding DNA sequence V3-V4 region universal primers 338F (5′- ACTCCTACGGGAGGCAGCA−3′) and 806R (5′- GGACTACHVGGGTWTCTAAT−3′) which contained a unique sequence tag to barcode each sample. PCR enrichment was performed in a 25μl reaction containing 12.5μl of 2 × Q5 Master Mix, 0.2μM of each primer,120ng of the extracted DNA, and Nuclease-free water. PCR reaction amplification conditions were: initial denaturation at 98°C for 5 mins; followed by 15-21 cycles of denaturation at 98°C for 10 s, primer annealing at 57°C for 30 s, extension at 72°C for 30 s; and a final extension step at 72°C for 5 min. The PCR products were purified with AmpureXP beads and eluted in the Elution buffer. Libraries were built with NEB Next UltraTM DNA Library Prep Kit for Illumina (New England Biolabs Inc, Ipswich, USA). And then the validated libraries were used for sequencing on the Illumina MiSeq platform (Illumina Corporation, San Diego, USA). The sequencing data have been deposited in NCBI BioProject PRJNA824624 with the Biosample accessions SAMN27409411-SAMN27409469.

### Bioinformatics analysis

The raw reads obtained by sequencing are filtered to obtain high-quality data (clean reads) for downstream analysis. Using the software FLASH (Magoč and Salzberg, 2011) (Fast Length Adjustment of Short reads,v1.2.11), the paired reads obtained by double terminal sequencing are assembled into a sequence, that is, a tag, by using the overlapping relationship. Use CUTADAPT (Martin, 2011) to remove tags containing primers, refer to the gold database (v20110519) chimera database, and use the UCHIME method in the VSEARCH (v2.3.4) (Rognes et al., 2016) software to remove the tags containing the chimera. Use VSEARCH (v2.3.4) software to cluster Tags with a similarity> 97% into an OTU, and get the OTU representative sequence. Use RDP classifier (v2.2) (Wang et al., 2007) software to compare OTU representative sequence with Silva(v128) database for species annotation. Alpha diversity is used to analyze the species diversity in the sample, using mothur (v1.39.5) (Schloss et al., 2009) software to calculate 5 indicators, including Chao, Ace, Shannon, and Simpson. Beta diversity is used to measure the diversity between samples, calculated using QIIME (v1.80) (Caporaso et al., 2010) software. The rest of the graphics are implemented using R package (v3.0.3). Use LEfSe (LDA Effect Size) (v1.0) (Segata et al., 2011) to calculate the LDA score value. The significant flora must meet the threshold *p* < 0.05 and the LDA score value ≥2.0 (or ≤ -2.0). Through the use of QIIME (v1.80) (Schloss et al., 2009) software, the use of similarity analysis (ANOSIM) for group comparison analysis, to find out the different components in the group.

### Machine learning process

Random forest analysis was used to perform classification. This method constructed multiple decision trees by using the information contained in input features and predicted the classification of three regions by combining multiple weak classifiers (Breiman, 2001). According to the random forest method in the R package RandomForest (v4.6-14), the OTU data of intestinal microorganisms in the three regions was used to build a model for predicting the sample distribution in the areas. The RF classification method was divided into two steps: one was to build a decision tree based on randomly selected samples (the training set) which include 70% of the original data set (42 samples). The other one was to use the test set which was the remaining samples (17 samples) in the original data set to verify the decision tree (Svetnik et al., 2003). In addition, the receiver operating characteristic (ROC) curve was used to evaluate the constructed model, and the area under the ROC curve (AUC) was used to designate the ROC effect to evaluate the potential of intestinal microbial markers to predict different regions.

## Results

### Correlation with age and sex of the subjects

The present study explored the relationship between the composition of the gut microbial community and age as well as sex in the entire population. The results of ANOSIM analysis of the present study based on Bray–Curtis distance showed that there was no significant difference in the gut microbial community between age 1 and age 2 group (*p* = 0.49), and the male and female group (*p* = 0.30).

### Whole sequencing data

**Figure 1:**
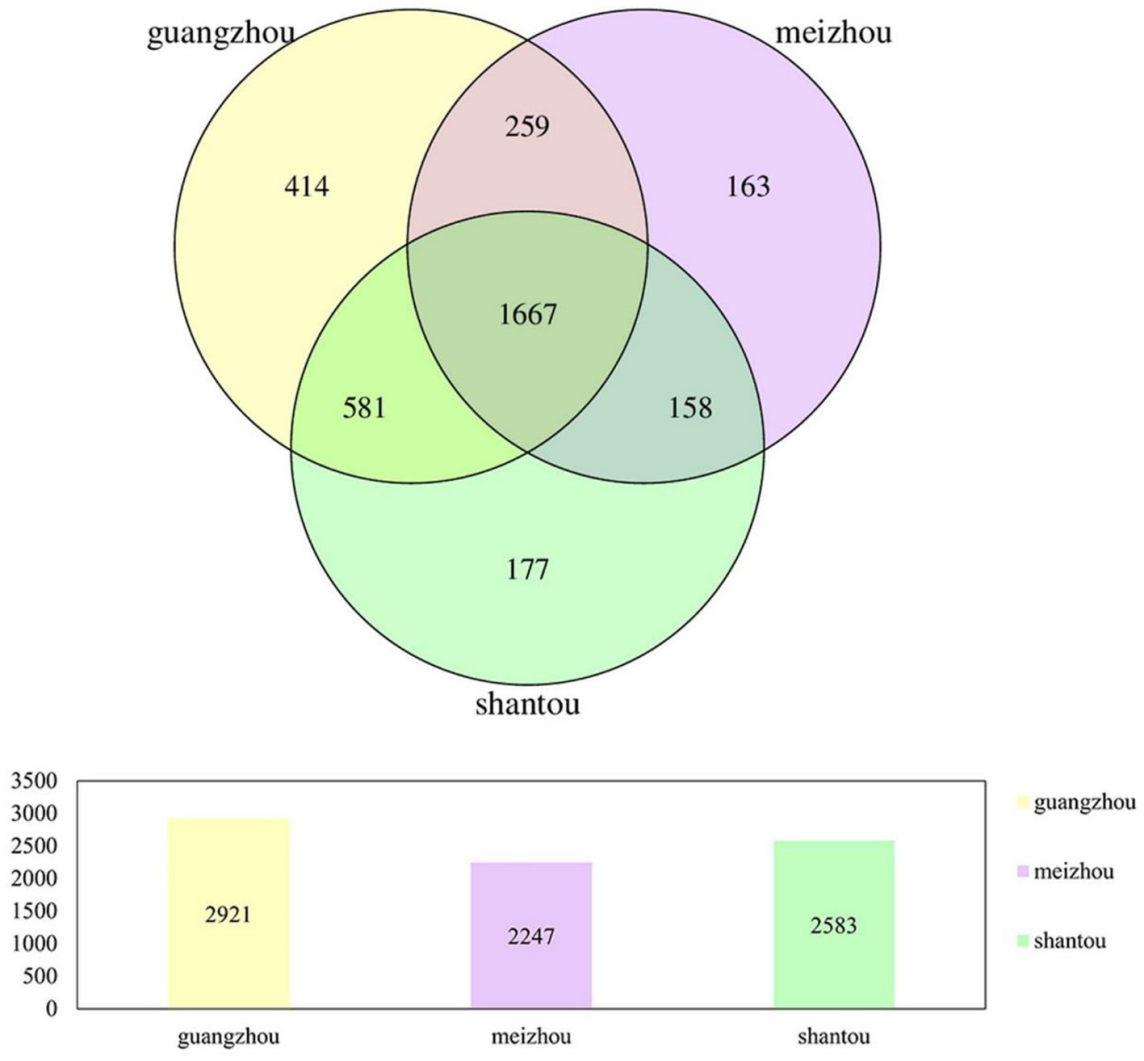
Venn diagrams of bacterial OTUs in all fecal samples from people in Guangzhou, Meizhou and Shantou.

Fecal samples of 59 healthy individuals from Guangzhou, Meizhou, and Shantou, Guangdong Province were subjected to high-throughput sequencing of 16SrRNA gene. After filtering, a data set consisting of 4256.44Mbp of effective and high-quality 16SrRNA gene sequences were generated, including 16,740,484 reads (median=221,912 reads, ranging from 79,892 to 599,496 reads; Supplementary Table 1). A cluster analysis of 97% similarity was performed to determine a total of 3,419 OTUs. All the valid sequences were annotated with species at different taxonomic levels, which yielded a total of 3,419 OTUs, belonging to 13 phyla, 15 classes, 21 orders, 35 families, 119 genera, and 22 species. The Venn diagram showed that the number of unique OTUs in Guangzhou, Meizhou, and Shantou was 414, 163, and 177, respectively, with 1667 OTUs shared by all the samples in the present study (Figure 1).

### Richness and diversity of microbial communities

**Figure 2:**
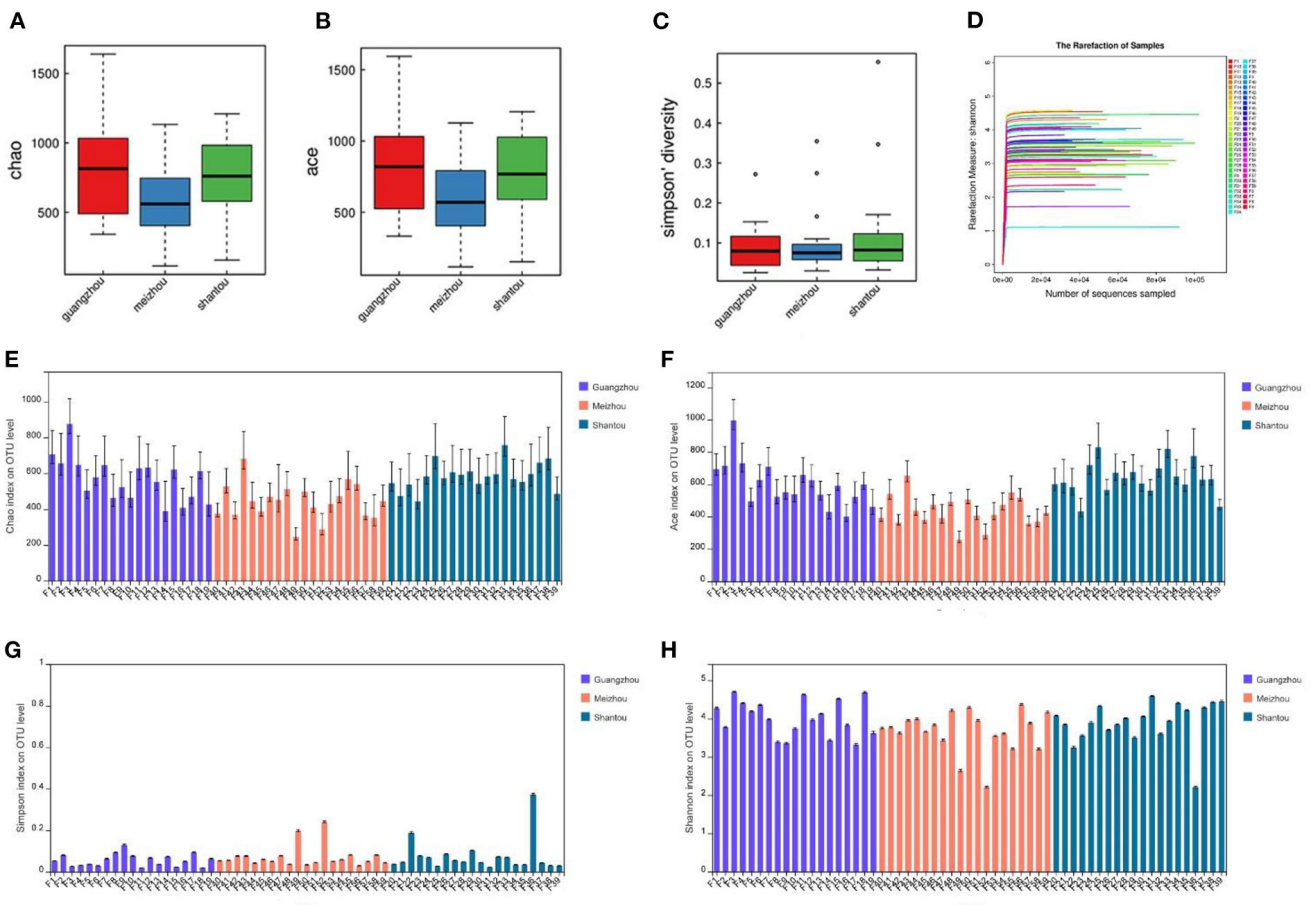
Differences in bacterial alpha diversity among the three regions: **(A,E)** Chao. **(B,F)** Ace. **(C,G)** Simpson diversity. **(D,H)** Shannon index.

Microbial complexity in the feces was estimated based on alpha-diversity indices (Chao, Ace, Simpson, and Shannon), and the results showed that there was no significant difference in the diversity among all individuals in each group (Figures 2E–H). Pairwise diversity of the three groups in the present study, the indices of Chao and Ace represented the species richness. The results of the present study showed that individuals from Guangzhou and Shantou had significantly higher index values as compared with those from Meizhou (Figures 2A,B). Results of the Simpson diversity index in the current study revealed that the three regions had similar statistical index values, indicating no significant difference in species diversity (Figure 2C; *p* > 0.05). In addition, the sparse curve of the Shannon index showed a trend toward saturation as presented in Figure 2D which illustrated sufficient sequencing depth.

### Overview of bacterial community composition

**Figure 3:**
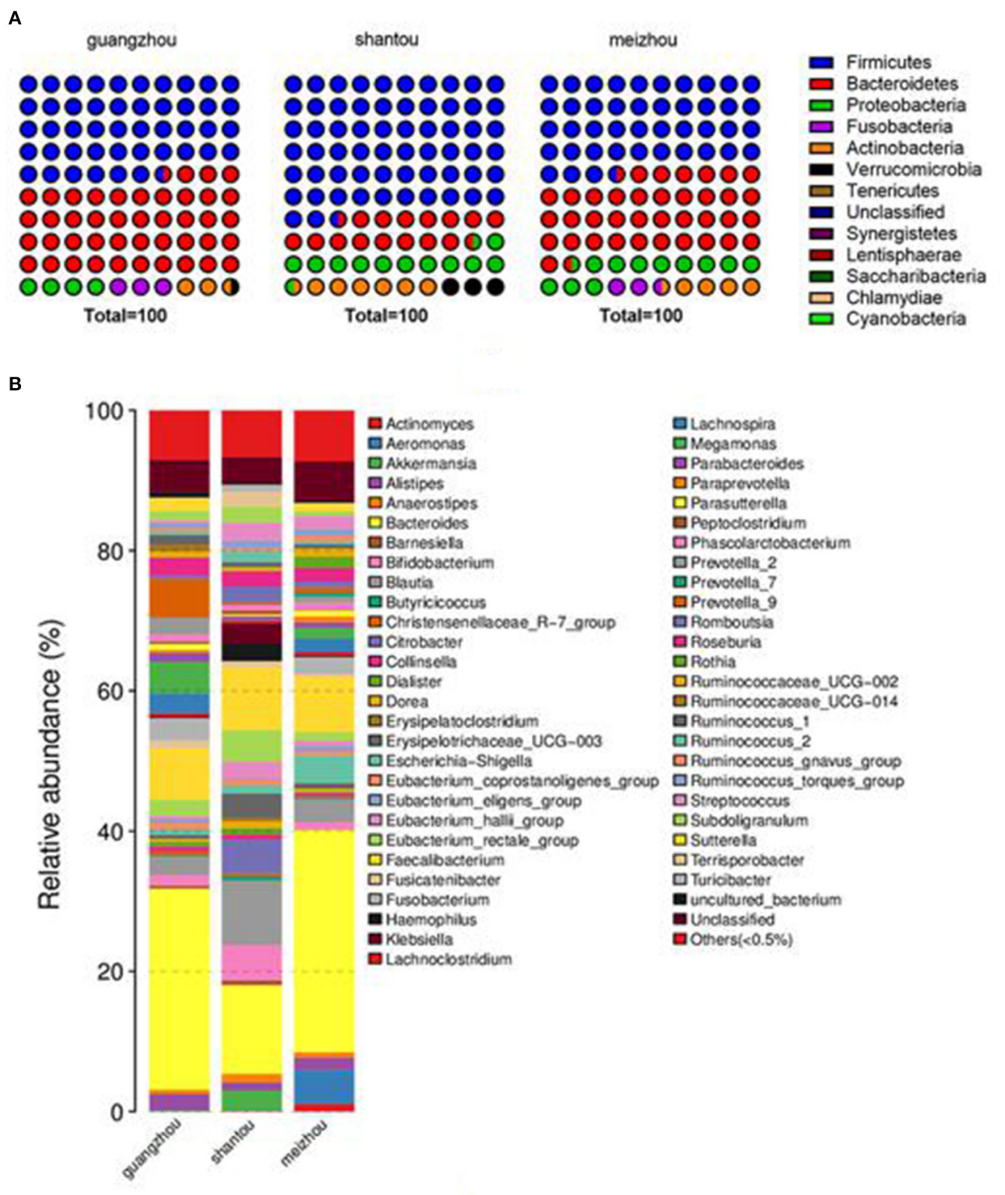
Distribution of intestinal microbes at different taxonomic levels in Guangzhou, Meizhou and Shantou populations. Two levels of dominant taxa are shown (Others: <0.5% relative abundance). **(A)** Distribution at the phylum level. **(B)** Distribution at the genus level.

The average relative abundance of the three groups at the phylum and genus level was also evaluated to further intuitively uncover the microbial composition characteristics in the three regional groups as presented in Figure 3. It was found that phylum Firmicutes was the most predominant phyla in Guangzhou, Meizhou, and Shantou, with relative abundances of 46.7, 43.4, and 62.5%, respectively. This was followed by phylum Bacteroidetes, which contributed 43.1, 38.2, and 16.1% of the total sequences. Further, it was noted that Bacteroides had the highest abundance in the bacterial communities of fecal samples at the genus level, accounting for 28.7, 31.7, and 12.7% in Guangzhou, Meizhou, and Shantou, respectively. On the other hand, Faecalibacterium accounted for 7.4, 7.9, and 8.9% in Guangzhou, Meizhou, and Shantou, respectively. The remaining top 10 bacterial genera were Blautia, Eubacterium_rectale_group, Bifidobacterium, Roseburia, Prevotella_9, Megamonas, Escherichia-Shigella, and Fusobacterium. Besides, it was found that the relative abundance of Bifidobacterium was 1.54%, 1.04%, and 5.09% in Guangzhou, Meizhou, and Shantou, respectively.

### Genus-level core intestinal flora and comparison of feces and saliva

The intestinal core microbiome was determined at the genus level and defined as bacteria with >0.1% abundance in ≥80% of the respective samples (Dehingia et al., 2015). It was found that there were six main genera in the fecal samples of all individuals, which constituted a genera-level phylogenetic core, including Bacteroides, Blautia, Eubacterium_hallii_group, Faecalibacterium, Lachnoclostridium, and Roseburia (Supplementary Table 2). Further, these fecal samples were used to compare with saliva samples we previously published (Yao et al., 2021) and the results of the comparisons were as shown in Supplementary Figure 1. The data of the present study on principal coordinate analysis (PCoA) based on genus-level abundance revealed that there was a clear distinction between fecal samples and saliva samples. Further, the linear discriminant analysis (LDA) histogram reflected that at the genus level, the relative abundance of Bacteroides, Faecalibacterium, Blautia, and Bifidobacterium was higher in the fecal samples, whereas the relative abundance of Streptococcus, Gemella, Porphyromonas, and Haemophilus was higher in the saliva samples.

### Beta diversity of bacterial communities

**Figure 4:**
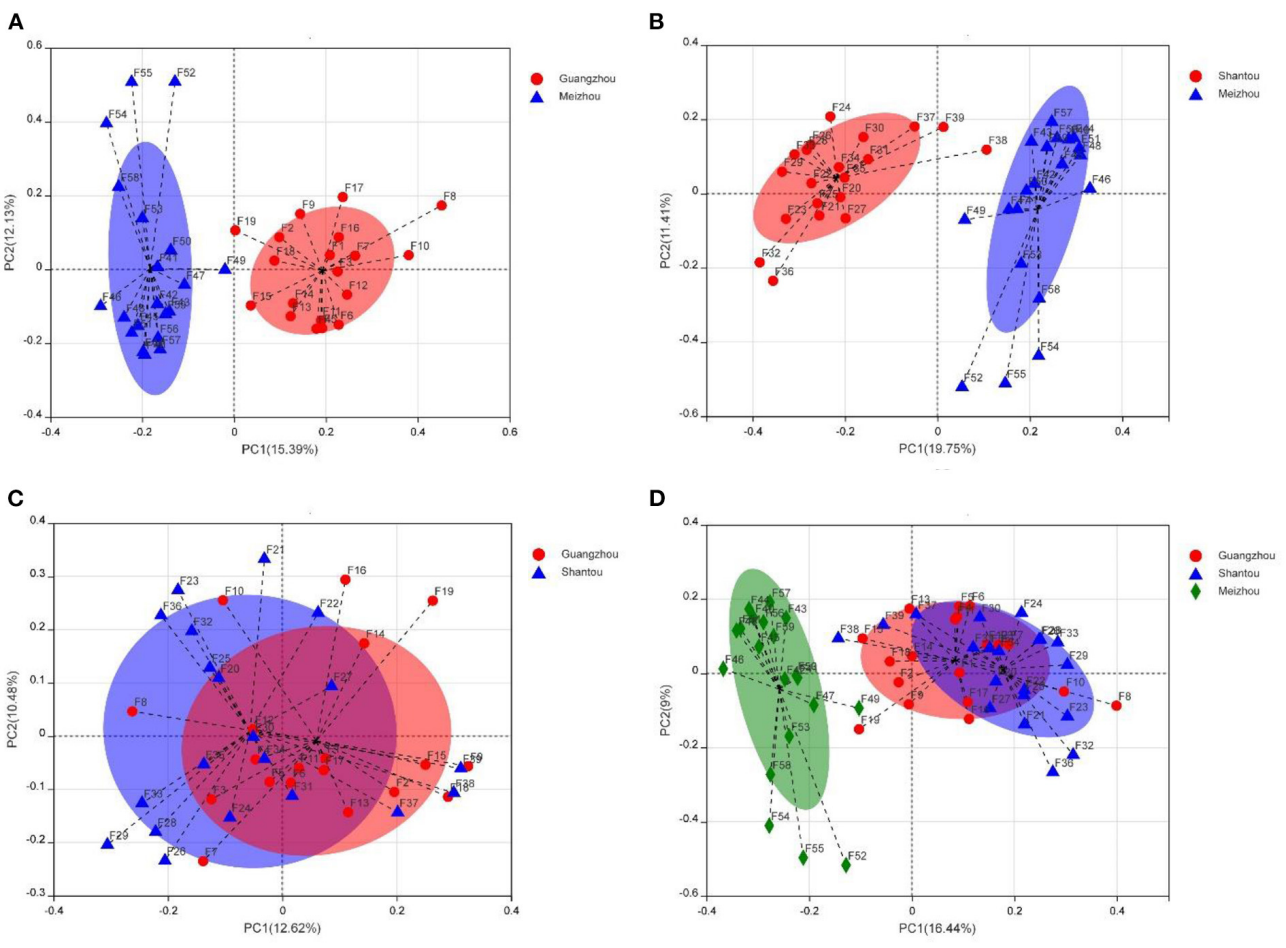
Taxonomic diversity of microbiomes from samples from Guangzhou, Meizhou, and Shantou. The principal coordinate analysis (PCoA) graph analysis is based on the Bray-Curtis distance at the operational classification unit (OTU) level, and each sample is represented by a point. **(A)** Guangzhou vs. Meizhou. **(B)** Shantou vs. Meizhou. **(C)** Guangzhou vs. Shantou. **(D)** Guangzhou vs. Shantou vs. Meizhou.

Beta diversity was assessed by PCoA and ANOSIM analysis using the Bray–Curtis distance method at the operational classification unit (OTU) level to further indicate the similarity between microbial communities. Although there were some slight overlaps in individual samples, the samples of Guangzhou and Meizhou groups, Shantou and Meizhou groups were roughly clustered. The similar structure of the intestinal microbiota community was found in the fecal samples between Guangzhou and Shantou, indicating an overlap in community structure (Figures 4A–C). The samples of the Meizhou population formed an “out-group,” which was generally not confounding with the samples of the Guangzhou or Shantou populations (Figure 4D). The ANOSIM analysis was performed on the three geographical groups (Supplementary Figure 2), and the results of this study demonstrated that the differences between the groups were greater than the differences within the groups, and the groupings were statistically significant (R = 0.3254, *p* = 0.0010).

### Comparison of differences among three regions

**Figure 5:**
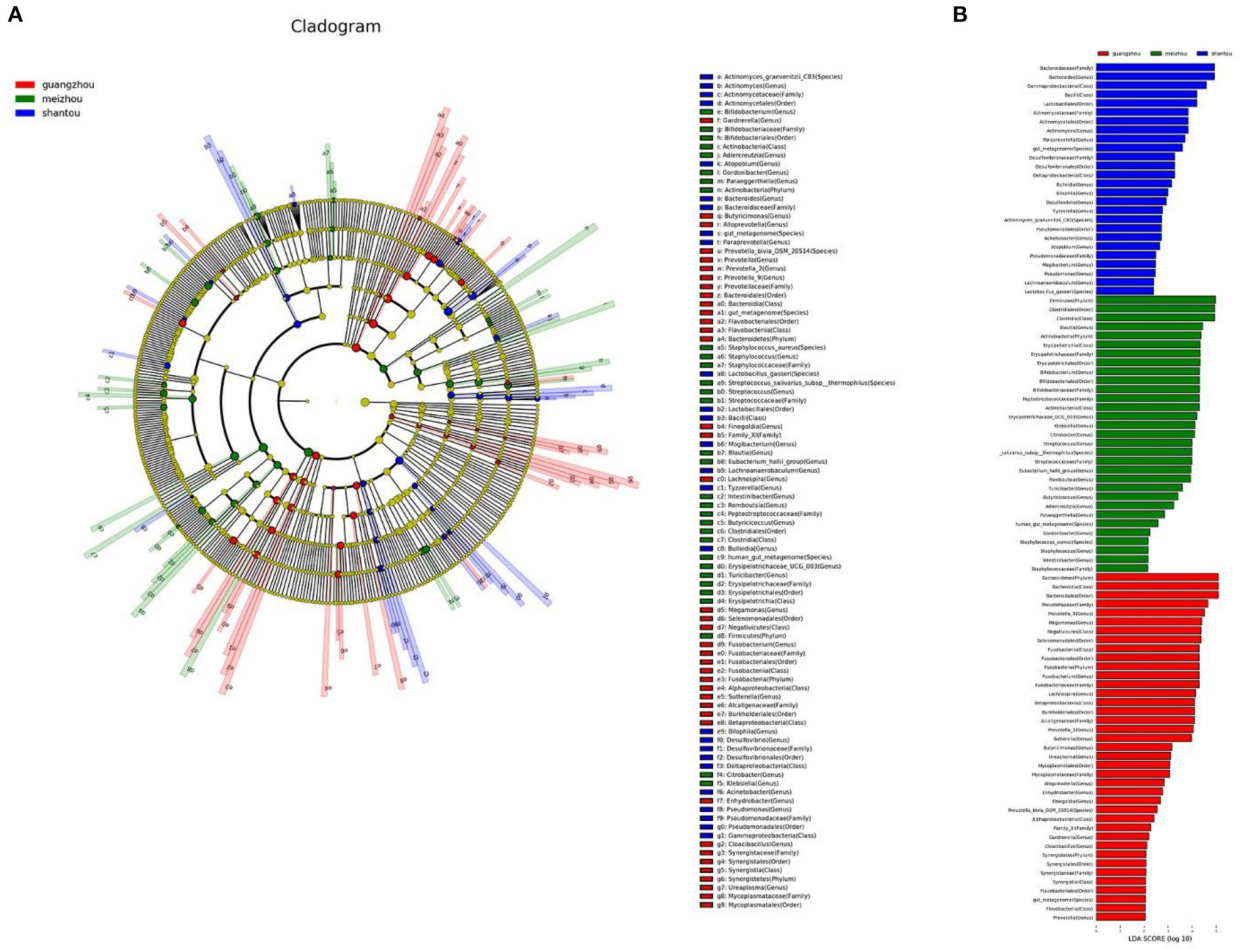
Differentially abundant taxa between the three regions. These different genera from phylum to genus were identified by linear discriminant analysis (LDA) using LEfSe. **(A)** Cladogram result graph. **(B)** Linear discriminant analysis (LDA) value distribution histogram. Red: Guangzhou; green: Meizhou; blue: Shantou.

The linear discriminant analysis effect size (LEfSe) test for biomarkers was used to find the taxa with the strongest effect on region differentiation. The Cladogram chart showed that there were at least two significant differences in the phylum, class, order, family, genus, and species level in the fecal samples from Guangzhou and Meizhou (Figure 5A). The composition of the microbial community of the fecal samples from Shantou at the phylum level was not significantly different from that of Guangzhou and Meizhou. In addition, at least three significantly different microorganisms were found at the level of class, order, family, genus, and species levels. Further, a total of 96 differentially abundant taxa were found in the three regions shown in the histogram of LDA value distribution (Figure 5B). At the phylum level, the significant differences in the samples of the Guangzhou and Meizhou populations were mainly Bacteroidetes, and Firmicutes, respectively. The top five microorganisms with significant differences at the genus level in the three regions included Prevotella-9, Megamonas, Fusobacterium, Lachnospira, and Prevotella_2 in Guangzhou, Bacteroides, Actinomyces, Paraprevotella, Bulleidia, Bilophila in Shantou, and Blautia, Bifidobacterium, Erysipelotrichaceae_UCG_003, Klebsiella, Citrobacter in Meizhou.

### Random forest

**Figure 6:**
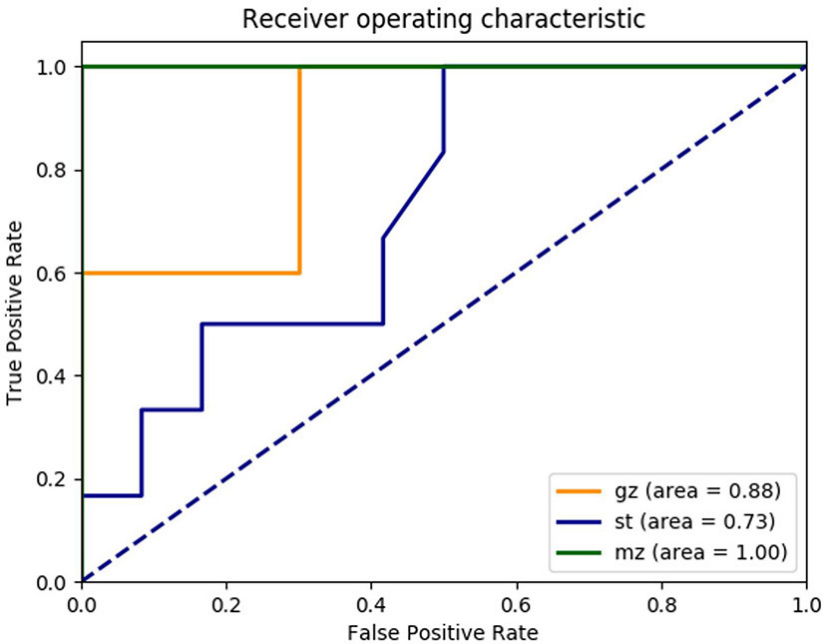
Receiver operating characteristic (ROC) curves trained on the OTU abundance demonstrate the performance of distinguishing fecal samples from Guangzhou, Shantou, and Meizhou. Yellow line: Guangzhou; blue line: Shantou; and green line: Meizhou.

During the construction of a random forest model based on the composition of gut microbes, top 230 OTUs markers were set as the best set. The markers performed well and were on the training set (*n* = 42, 14 samples in Guangzhou, Shantou and Meizhou). The validation set of the random forest model (*n* = 17, 5 Guangzhou samples, 6 Meizhou samples, and 6 Shantou samples) showed that 12 of the 17 validation samples were correctly classified, and 100% of the Meizhou samples were correctly predicted, whereas 2 Guangzhou samples (F7 and F8) were identified as Shantou samples and 3 Shantou samples (F23, F25, and F34) were identified as Guangzhou samples, with an overall accuracy of 70.59%. The performance of the model was evaluated using ROC analysis. The AUC of the area under the curve in Guangzhou, Shantou, and Meizhou were 0.88, 0.73, and 1.00, respectively (Figure 6).

## Discussions

The present study explored the correlation between the gut microbiota of the entire population and age as well as sex. Further, the ANOSIM analysis showed that there were no statistical difference between the intestinal microbial community structures between 16 and 32 as well as between 33 and 62 years of age. Previous studies had shown that Bifidobacterium was dominant in infants and a larger proportion of Bacteroides was dominant in elderly individuals (Claesson et al., 2011; Yatsunenko et al., 2012). On the other hand, Firmicutes and Bacteroidetes as the dominant bacteria were mainly dominant in adults. The established microbiota composition remained unchanged when there was no change in long-term eating habits and pathophysiology (Adak and Khan, 2019). In the current study, the small difference between the two age groups could be associated with most young individuals in the current study (45 cases, 76.27% between 25–45 years old), with only one individual who was over 60 years old. In addition, it was evident from the results of this study that there were no statistical differences in fecal microbiota between males and females. The finding of the present study was consistent with the results of a study carried out by Arumugam et al. that found that sex had no effect on the structure of the gut microbes of individuals from six different countries (Arumugam et al., 2011). Moreover, several other studies have also shown that sex factors have less influence on the gut microbial community than other factors (Kovacs et al., 2011; Human Microbiome Project Consortium, 2012).

The analyses performed at the phylum level in the present study showed that the intestinal microbiota of this research was made up of the four most important phyla, including Firmicutes, Bacteroidetes, Proteobacteria, and Actinobacteria. It was evident that phylum Firmicutes and Bacteroides were the most abundant. This was similar to the results of previous studies (Jandhyala et al., 2015). Although the diversity of gut microbes at the phylum level was low, it was noted that they had significantly high diversity at the genus level. From the results of the current experiments, the predominant genera in all individuals was Bacteroides, followed by Faecalibacterium. A previous study reported that China had the highest abundance of Bacteroides at the genus level as compared with four other countries. This was consistent with the findings of the present study. Furthermore, the study reported that Japan had higher levels of Bifidobacterium whereas the abundance of Prevotella and Faecalibacterium was relatively higher in Korea (Nam et al., 2011). Previous studies had also indicated that Faecalibacterium was more dominant in the populations of Hadza, Italy, and the United States. Furthermore, Prevotella was a significant genus found among the Indian tribes, Mongolians, American Indians, and Malawi tribes (Dehingia et al., 2015). This difference in dominant genus originates from the variations in the intestinal microbiome, whereas the changes in the intestinal microbiome may be caused by geography and ethnicity among other factors (Dwiyanto et al., 2021).

One of the main interests of human gut microbial research was toward the core microbiota. The bacterial genera of Faecalibacterium, Eubacterium, Clostridium, Blautia, Ruminococcus, and Roseburia were found to be the core gut microbiota in the representative populations of the world (Dehingia et al., 2015). In the current study, six genera-level core intestinal bacteria of the gut microbiota, ubiquitously in unrelated individuals from Guangdong, which were Bacteroides, Blautia, Eubacterium_hallii_group, Faecalibacterium, Lachnoclostridium, and Roseburia. A microbial analysis report from nine provinces in China revealed a total of nine core bacteria (Balutia, Clostridium, Ruminocossus, Faecalibacterium, Subdoligranulum, Roseburia, Coproccus, Bacteroides, and Phascolarctobacterium) (Zhang et al., 2015). In healthy western individuals, Bifidobacterium, Bacteroides, Faecalibacterium, Ruminococcus, Blautia, Dorea, Eubacterium, and Coprococcus were the core intestinal bacteria genus (Martínez et al., 2013). Further, the intestinal core flora shared by these people were Bacteroides, Blautia, and Faecalibacterium. In addition, more than 45% of the common bacterial genera could be detected in both feces and oral cavities (Segata et al., 2012). It is worth mentioning that the establishment of the intestinal saliva microbial communities was similar. According to a study by Schmidt et al., transmission to, and subsequent colonization of the large intestine by oral microbes commonly occurred in healthy individuals. Although it has been previously reported that *Streptococcus salivarius* and *S. mutans* were particularly found in saliva (Tagg and Ragland, 1991). A study conducted by Kai-NanZou et al. showed that the bacteria in the intestines overlapped with those in feces (Zou et al., 2016). These results indicated that the identification of sample types using a single microbial marker may be misjudged. The findings of fecal samples in the present study were compared with those of saliva samples in our previously published study (Yao et al., 2021). In addition, the results showed that fecal and saliva samples can be distinguished, which could avoid the defect of single microbial markers to identify both saliva and feces samples.

The PCoA displayed regional differences in intestinal microorganisms between Meizhou and the other two regions. Different geographic origins of humans may result in diverse compositions of the gut microbiome, due to distinctive genetic backgrounds or life environments (Li and Zhao, 2015). Guangfu and Chaoshan populations occupied the two rich areas of the Pearl River delta plain and Chaoshan plain, respectively. The barren and backward mountainous areas of northern and eastern Guangdong were the basic distribution areas of the Hakka people. Several studies have demonstrated that geographic location plays an important role in shaping the intestinal microbial community, and dietary habits could also affect the composition and distribution of intestinal microbes (De Filippo et al., 2010; Zhang et al., 2013; Singh et al., 2017). Through a return visit to the volunteers in the three regions, they simply recorded their eating habits. The Meizhou area was dominated by greasy food, whereas the Guangzhou and Shantou areas were dominated by intake of a light diet (Song et al., 2005; Zhong et al., 2017; Wang et al., 2019). A high-fat diet had been shown to reduce the diversity and richness of human gut microbial communities, which was negatively correlated with the abundance of Bifidobacterium. Furthermore, Caesar et al. reported that Bacteroides increased in mice fed with lard (De Filippo et al., 2010; Caesar et al., 2015; Khine et al., 2019). The results of the present study suggested that the intestinal microbes in Meizhou had the lowest abundance of Bifidobacterium and microbial alpha diversity, whereas Bacteroides showed the highest abundance among the three regions. This might be related to the fact that Hakka ancestors lived in mountainous areas with inconvenient transportation, expended much physical strength on their daily labor, and needed to supplement foods with rich fat sources such as lard, developing a diet that preferred greasy foods. Therefore, diet may also be an important factor affecting the microbial differences in fecal samples from the three geographical regions. The dietary associations seen here paralleled a recent study comparing European and African, Europeans consuming high-fat foods formed a typical taxonomy dominated by Bacteroidetes, while Africans consuming low-fat diets had higher microbial diversity (De Filippo et al., 2010). At the same time, a study of American populations showed that the gut flora of individuals with a typical western diet high in animal fat and protein was dominated by Bacteroides (Wu et al., 2011). There are, of course, many differences between the three regions that might influence the gut microbiome, but dietary differences provide an attractive potential explanation.

According to the results of the ANOSIM analysis, there were significant differences in the intestinal bacterial community composition in samples from the three regions. A previous study identified three intestinal types: Bacteroides (Enterotype 1), Prevotella (Enterotype 2), and Ruminococcus (Enterotype 3) (Arumugam et al., 2011), which could afford a strong discriminatory classification ability among European individuals, although other studies had reported that Enterotype 3 was an uncertain bacterial composition (Liang et al., 2017). Hyun Seok et al. showed that structure of gut microbiota variations across the geographical location. The characterization of population distribution according to the three enterotype classifications showed that the distributions of Enterotype 2 and Enterotype 1 differed by region. Samples from the U.S. and Japan had large numbers of Enterotype 1, while samples from Amazon natives in Venezuela, as well as from Malawi and Tanzania in Africa had large numbers of Enterotype 2 (Oh et al., 2022). In the present study, linear discriminant analysis (LDA) using LEfSe showed that Shantou, Guangzhou, and Meizhou belonged to Enterotype 1, Enterotype 2, and Enterotype 3, which were mainly composed of Bacteroides, Prevotella, and Blautia, respectively.

The present study attempted to construct a prediction model on the basis of OTU abundance of a genus of intestinal microbes for biogeographic inference. According to the parameter importance ranking of random forest, the most important characteristic differences in classification were mainly Bacteroides, Lactobacillus, and Prevotella-9. Similar to LEfSe analysis, it might be inferred that the main flora of intestinal microbes could be used as a factor in predicting geographic location. Likewise, a study conducted by De Filippo et al. found that Firmicutes and Bacteroides could distinguish children in rural Europe and Africa has significantly demonstrated that Prevotella was a powerful tool for discriminatory classification (De Filippo et al., 2010). The present study found that through verification, the accuracy of the predictions in the three regions was very high, especially in the Meizhou area, where the AUC was 1. All the samples from Meizhou in the verification set were correctly classified, whereas the performance of Guangzhou and Shantou was not satisfactory (the Guangzhou sample and the Shantou sample misjudged each other). Further, the finding of this study was similar to the results of PCoA. It might be possible that a combination of geography, dietary, and other factors play an important role (Yatsunenko et al., 2012). This needs to be understood by further research.

This study provides the first insight into the gut microbiome data of the three characteristic Han populations in Guangdong, which can enrich gut flora information of Chinese ethnic groups. And joint analysis of geography and diet might be helpful to provide enlightening information for forensic science. In addition, due to the complexity of the population composition and living environment of Guangdong Province, so the representativeness of researching samples from the selected three regions is limited. In our current study, individual differences need to be analyzed with large sample size, and the research is still limited to the relative abundance at the genus level. In the future, the sample size will be expanded, sample table information will be recorded in detail (recording used water sources, Food Frequency Questionnaire (FFQ), and other factors), and fecal microbiome analysis will be performed in depth based on microbial species level and sequence. In order to observe the flora differences in different regions of Guangdong Province, follow-up studies will further explore the gut flora of multi-ethnic and multiregional populations.

## Conclusion

In conclusion, the current study used high-throughput sequencing methods to study the characteristics of the fecal microbial community of healthy Han individuals living in three regions of Guangdong Province. The results of the current study showed that the composition of intestinal microbes was mainly composed of Bacteroides, Faecalibacterium, and Blautia at the genus level. The feces could be significantly distinguished from saliva samples according to microbial differences at the genus level of both. Further, the populations in the three regions exhibited different enterotype classifications and the prediction model based on the random forest algorithm evidently showed a significant effect in distinguishing individuals, which might be due to regional differences. In conclusion, microbial community information in feces may have the potential for forensic analysis of body fluid traceability and regionally specific.

## Data availability statement

The datasets presented in this study can be found in online repositories. The names of the repository/repositories and accession number(s) can be found below: https://www.ncbi.nlm.nih.gov/, SAMN27409411-SAMN27409469.

## Ethics statement

The studies involving human participants were reviewed and approved by Biomedical Ethics Committee of Southern Medical University, Guangzhou, China. Written informed consent to participate in this study was provided by the participants' legal guardian/next of kin.

## Author contributions

LH: conceptualization, methodology, visualization, investigation, writing—original draft. LD and CL: validation, formal analysis. EH, XH, CX, and XL: resources, supervision, data curation. HS, CL, and LC: writing—review & editing. All authors discussed the results and contributed to the final manuscript.

## Funding

This project was supported by the Open project of Natural Science Foundation of Guangdong Province (Grant no. 2020A1515010938), Science and Technology Program of Guangzhou, China (Grant no. 2019030016 and Grant no. 202102080308), and Medical Science and Technology Research Foundation of Guangdong Province (A2019443). We are grateful to all volunteers who contributed samples for this study.

## Conflict of interest

The authors declare that the research was conducted in the absence of any commercial or financial relationships that could be construed as a potential conflict of interest.

## Publisher's note

All claims expressed in this article are solely those of the authors and do not necessarily represent those of their affiliated organizations, or those of the publisher, the editors and the reviewers. Any product that may be evaluated in this article, or claim that may be made by its manufacturer, is not guaranteed or endorsed by the publisher.
