# Supplementary material for: Fecal microbial signatures of healthy Han individuals from three bio-geographical zones in Guangdong
Source: Front Microbiol. 2022 Aug 8;13:920780. doi: 10.3389/fmicb.2022.920780 (PMC9393523; doi:10.3389/fmicb.2022.920780)
Supplement: Supplementary file 1 [file Data_Sheet_1.docx]

**Supplementary Table 1:** Sequencing statistics of the 16S rRNA gene V3-V4 region.

**Supplementary Table 2:** Dominant taxa at the genus level and their relative abundance in all samples. Grey filled cells indicate the core gut bacterial genera.


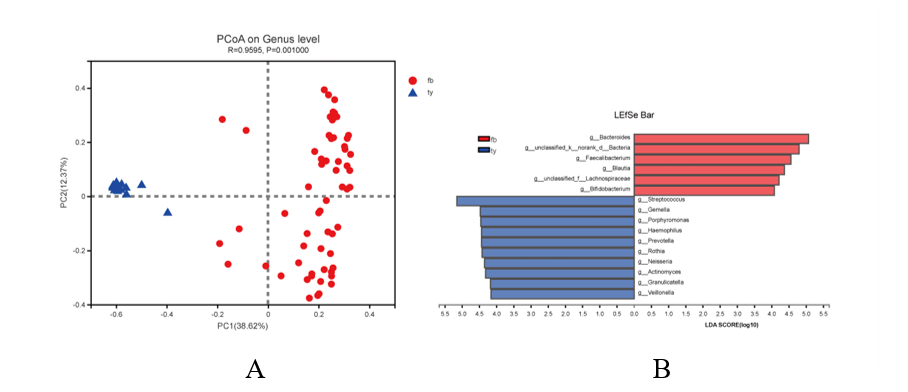


**Supplementary Fig. 1:** Distinguishing between fecal samples and saliva samples. **A** The principal coordinate analysis (PCoA) graph. **B** linear discriminant analysis (LDA) value distribution histogram. Red (fb): fecal samples; blue (ty): saliva samples.


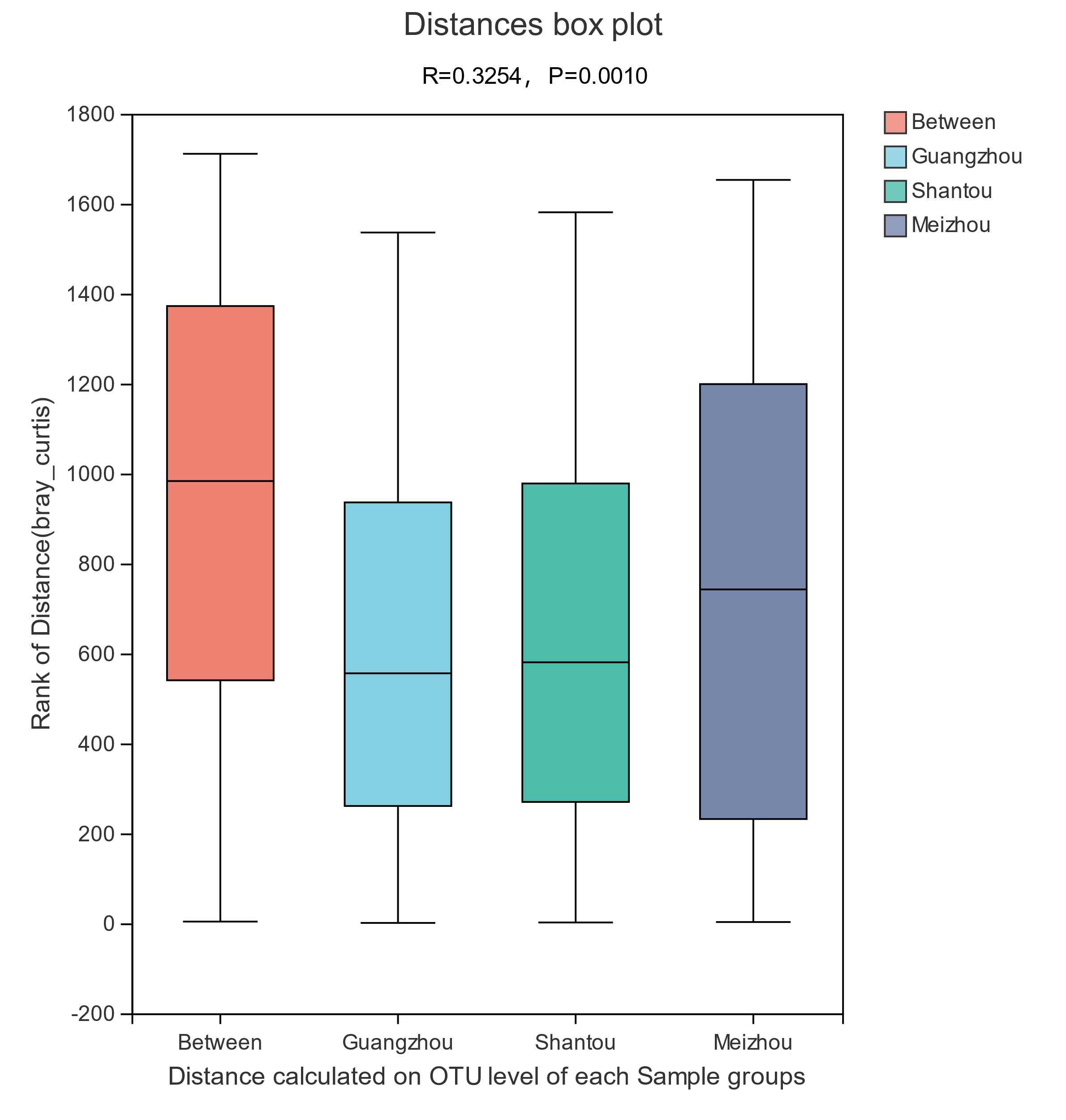


**Supplementary Fig. 2:** Analysis of between-group and within-group differences. The box extends from 25th percentile to 75th percentile, with a line at the median. Red filled box: Between-group; light blue filled box: Guangzhou group; green filled box: Shantou group; dark blue filled box: Meizhou group.
